# Essentially Leading Antibody Production: An Investigation of Amino Acids, Myeloma, and Natural V-Region Signal Peptides in Producing Pertuzumab and Trastuzumab Variants

**DOI:** 10.3389/fimmu.2020.604318

**Published:** 2020-12-07

**Authors:** Wei-Li Ling, Chinh Tran-To Su, Wai-Heng Lua, Jun-Jie Poh, Yuen-Ling Ng, Anil Wipat, Samuel Ken-En Gan

**Affiliations:** ^1^ Bioinformatics Institute, Agency for Science, Technology and Research (A*STAR), Singapore, Singapore; ^2^ Newcastle Research and Innovation Institute (NewRIIS), Singapore, Singapore; ^3^ Experimental Drug Development Centre, Agency for Science, Technology and Research (A*STAR), Singapore, Singapore; ^4^ School of Computing, Newcastle University, Singapore, Singapore

**Keywords:** signal peptide, antibody leaders, myeloma, antibody families, recombinant production, essential amino acid, non-essential amino acid, logistic regression

## Abstract

Boosting the production of recombinant therapeutic antibodies is crucial in both academic and industry settings. In this work, we investigated the usage of varying signal peptides by antibody V-genes and their roles in recombinant transient production, systematically comparing myeloma and the native signal peptides of both heavy and light chains in 168 antibody permutation variants. We found that amino acids count and types (essential or non-essential) were important factors in a logistic regression equation model for predicting transient co-transfection protein production rates. Deeper analysis revealed that the culture media were often incomplete and that the supplementation of essential amino acids can improve the recombinant protein yield. While these findings are derived from transient HEK293 expression, they also provide insights to the usage of the large repertoire of antibody signal peptides, where by varying the number of specific amino acids in the signal peptides attached to the variable regions, bottlenecks in amino acid availability can be mitigated.

## Introduction

The signal peptide (SP) of a protein is a short tag of amino acids at the N- or C-terminal that predestinates the protein location extracellularly or within the cell to the organelles. Known organelle targeting SPs include the nucleus localization (1) or export signal (2), mitochondria signals (3), endoplasmic reticulum (ER) secretion (4) or retention signal (5), and peroxisome signals (6).

Secreted by plasma B cells, antibodies are tagged with the ER secretion signal/SP at the N-terminal and translocated into the ER lumen (7), before being passed to the Golgi apparatus and sorted into secretory vesicles for extracellular secretion (8). The SP is unique to the protein and generally contains a positively charged N-terminal, followed by a hydrophobic region and a neutral polar C-terminal (9). Depending on its location at the protein N- or C-terminus, a cleavage site recognized by a signal peptidase (10) separates the SP from the protein. While secretory SPs are involved in the co-translational co-translocation pathway (4) with the primary function to export proteins (11), it remains enigmatic to why antibodies utilize a large repertoire of SPs (e.g. Vκ1 family with 22 SPs, VH3 family with 50 SPs, retrieved from the IMGT database (12) at the point of writing), hinting of their possible contributing roles in antibody production.

To boost production, modifying SPs has been largely successful in many studies (13–17, 18–21), however, the underlying mechanism of such effects remains enigmatic, e.g. limited understanding to the utilization of the huge antibody SP repertoires. With recent studies demonstrating cross-talks of antibody elements, where the constant regions (22, 23), variable regions and their pairings (24, 25) affect antibody production and function, there is increasing evidence that antibodies ought to be investigated holistically (26), especially when gearing towards better therapeutics (27). Through the inclusion of antibody SP in the analysis, deeper insights into antibody V-region pairing effects in transient recombinant production (24) can be further holistically considered. In this work, we unraveled the role of total amino acid usage that may underlie the usage of the diverse repertoire of antibody SPs in compensating for V-region hypervariability to overcome production bottlenecks.

## Materials and Methods

**Figure 1:**
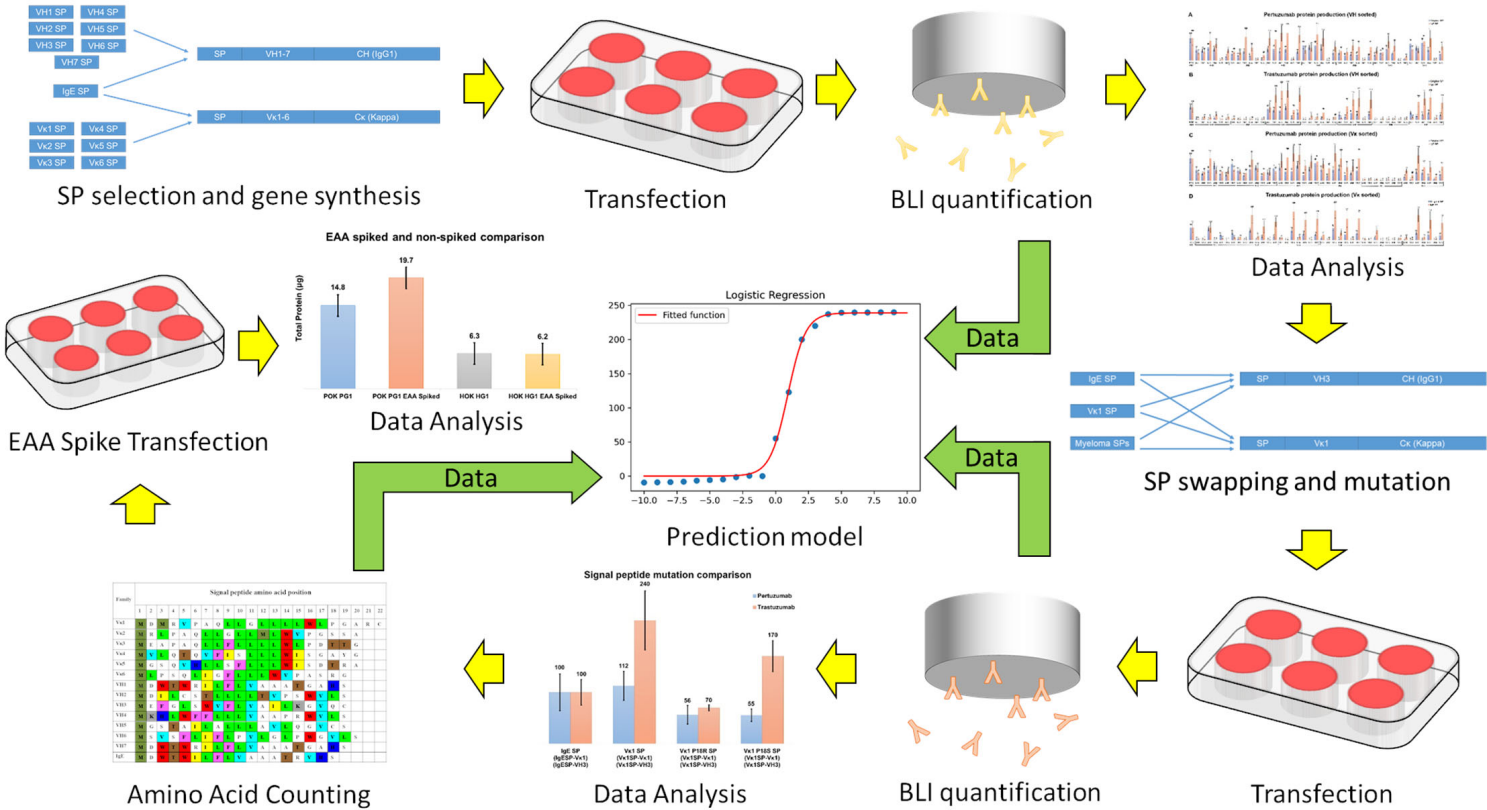
A schematic of workflow in the present study. Yellow arrows indicate experiment sequences while green arrows indicate data flow for constructing the predictive model.

The overall workflow is presented in **Figure 1**.

### Signal Peptide Selection

SP data were retrieved from the IMGT database for the respective light and heavy chain families. Consensus sequences within each family SP were determined using WebLogo (28) (https://weblogo.berkeley.edu/logo.cgi) to derive the VH and Vκ family SPs (**Supplementary Figures 1** and **2**). At the time of writing, Vκ4, Vκ5 and VH7 have 1 sequence each and VH5 and VH6 have 2 sequences each. Due to this lack of repertoire within its respective family, Vκ4, Vκ5 and VH7 SP sequence is used as it is while VH5 and VH6 were chosen based on similarity among heavy chain families.

### Recombinant Antibody Production

All VH and Vκ sequences were described previously (24) and codon optimized using GenSmart™ Codon Optimization (Genscript) online software, followed by synthesis by Eurofin (Japan) to avoid codon usage bias. The genes were transformed into competent *E. coli* (DH5α) strains (29) followed by plasmid extraction (Biobasic Pte Ltd) and sub-cloned into pTT5 vector (Youbio) using restriction enzyme sites, as previously performed (22, 24, 25, 30).

For testing the effects of additional essential amino acid (EAA) in boosting production, Impact EAA (Myprotein^TM^) were dissolved in water and filtered with 0.2um filter before supplementing the cell culture media following standard transfection protocol. The supernatant were harvested after 14 days and tested for production levels.

### Site Directed Mutagenesis and SP Swapping

Mutations in Vκ1 SP were performed using QuikChange Lightning Site-Directed Mutagenesis Kit (Agilent) with the respective primers: P18S forward: 5′- CTG CTG CTG CTT TGG CTT TCT GGC GCT AG-3′; P18S reverse: 5′-CTA GCG CCA GAA AGC CAA AGC AGC AGC AG-3′; P18R forward: 5′- GCT GCT TTG GCT TCG TGG CGC TAG ATG CG-3′; P18R reverse: 5′- CGC ATC TAG CGC CAC GAA GCC AAA GCA GC-3′ following protocols according to manufacturer recommendations.

SP graftings were performed using overhanging primers to the various V-genes via PCR using Q5 polymerase (NEB) with the following primers: VH3 18P 1st leader extension forward: 5′-CAG CTG CTG GGC CTG CTG CTG CTT TGG CTT **CCT** GGC GCT AGA TGC GAA GTG CAG CTG GTG-3′; VH3 18R 1st leader extension forward: 5′-CAG CTG CTG GGC CTG CTG CTG CTT TGG CTT **CGT** GGC GCT AGA TGC GAA GTG CAG CTG GTG-3′; VH3 18S 1st leader extension forward: 5′- CAG CTG CTG GGC CTG CTG CTG CTT TGG CTT **TCT** GGC GCT AGA TGC GAA GTG CAG CTG GTG-3′; VH3 18P 2nd leader extension forward: 5′- GCC GAA TTC GCG GCC GCG TTC CTC ACC ATG GAC ATG AGA GTT CCA GCT CAG CTG CTG GGC-3′; VH3 leader extension reverse: 5′-GCC GCG AAG CTT TCA CTT GCC AGG AGA-3′.

### Bio-Layer Interferometry Quantification

The Octet Red96 system (ForteBio) was used to quantify the amount of antibodies in transiently co-transfected cell cultures supernatants using Protein G biosensors (ForteBio) with preloaded program settings (high sensitivity assay with regeneration) in Octet Data Acquisition v10.0 as previously described (22, 24, 25, 30).

Quantification data were analyzed using Octet data analysis v10.0 with protein standard ranged from 100 μg/ml to 0.1953 μg/ul in two-fold serial dilution as per described in (24, 30).

### Constructing the Statistical Predictive Model

The logistic regression model was constructed using the data of the present and previous antibody production levels (24), totally N=168 samples. The dataset was randomly distributed into model set *M* (n=118) and two independent test sets *T_a_* (n=30) and *T_b_* (n=20). The prediction was evaluated using the two test sets T_a_ and T_b_ that was not used for model training and hence “unseen” by the model. The processes of training and testing were performed in triplicates.

Production rates were categorized into low (< 20%), medium (from 20% to 70%) and high (> 70%) production normalized by datasets of the IgE SP. The Logistic Regression CV classifier using “lbfgs” optimizer with L2 norm [implemented in the scikit learn v.0.22.1 package (31)] was used in a 20-fold cross validation process (e.g. using the model set *M*, of which 95% samples for training and 5% samples for validation, and repeating 20 cycles) to fine-tune the regularization strength. A weighted precision scoring function was used to evaluate the model performance and the categorized classes (low, medium, high) were weighted to counter the slight imbalance in the dataset, i.e. 65% low, 70% medium, and 33% high production samples. The optimization process was performed in 1000 iterations. The probability of each categorized production label (class label) of each antibody variant was calculated using the equation below:

$$P\left(y_{k_{i}}\right)=\frac{1}{1+e^{-\left(\beta_{0_{k}}+\beta_{k_{i}}\cdot X_{i}\right)}}$$

where:

$P\left(y_{k_{i}}\right)$ is probability of the predicted class label *y_k_* (i.e. the production level) of variant *i*, with *k* = 0 (low), 1 (medium), 2 (high)

$\beta_{0_{k}}$ is the corresponding intercept of class k, e.g. β_0_ = [−17.88, 14.62, 3.25] with respect to *k* = [0, 1, 2]

$\beta_{k_{i}}$ is array of regression coefficients of features (amino acid contribution) in variant *i* for each class *k*

*X_i_* is array of amino acid counts in each variant *i*

To evaluate the model performance, average area under ROC (AUC) of all pairwise combinations of classes and averaged F1-score were computed on the testing sets. In addition, a dummy model using Dummy classifier with default parameters was created and used as a baseline control.

*The script is available and provided upon request.

## Results

### “IgE Signal Peptide” Results in Better Antibody Production Rates

Antibody SPs were initially named with respect to the constant region isotypes but were recently re-classified in IMGT by the V-region family. With exception to the wild-type IgE signal peptide: Humighae 1 (Genbank accession J00227), termed “IgE” SP, references to the SPs in this research follow the IMGT convention. Given the large number of antibody germline SPs, we selected consensus/dominant representatives of each VH and Vκ family as representative “native” SPs.

VH and Vκ variants of Pertuzumab and Trastuzumab CDRs were paired with their respective SPs (i.e. VH1 SP to VH1 framework (FWR), Vκ1 SP to Vκ1 FWR) and the production levels compared to utilizing only the IgE SP from our previous work (24). Given the variability in transient co-transfections, recombinant Pertuzumab and Trastuzumab Vκ1 VH3 with the IgE SP were used for normalization (100%) to facilitate comparisons. The high CDR similarity between both Trastuzumab and Pertuzumab (24) allowed the isolation of effects to be due to SP as well as for analyzing the impact of minute CDR differences on protein production.

**Figure 2:**
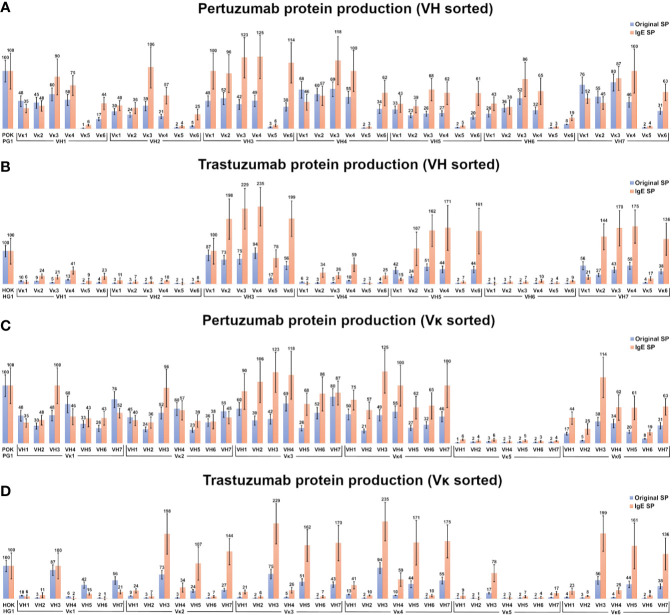
Production rates (%) of recombinant antibodies containing various heavy and light chain pairings. **(A, B)** Recombinant Pertuzumab **(A)** and Trastuzumab **(B)** variants with the various SPs grouped according to VH families. **(C, D)** Recombinant Pertuzumab and Trastuzumab variants with the various SPs grouped according to Vκ families. Recombinant Pertuzumab and Trastuzumab variants produced with native (blue) or the ’IgE’ (orange) SPs respectively, are shown. In all experiments, the recombinant wild-type Pertuzumab (POK PG1) or Trastuzumab (HOK HG1) was used (shown in the first column) for normalization. The production rate of the variants utilizing the ‘IgE’ SP (23) was used as the reference for the production rate.

Within the Pertuzumab variants (**Figures 2A, C**), production levels of antibodies with the IgE SP (24) tend to be higher than those using native SPs (e.g. mean antibody production using the IgE SP at 56 %, while antibody utilizing their respective SP, were at 34 %) with exceptions (Vκ1|VH1, Vκ2|VH1, Vκ1|VH4, Vκ2|VH4, Vκ1|VH7, and Vκ2|VH7). Regardless of the SP, antibodies paired with Vκ5 family constructs gave poor yields (**Figure 2C**, sorted by light chain families).

Of the Trastuzumab variants (**Figures 2B, D**), there was a general agreement to the trends observed in the Pertuzumab dataset where IgE SP variants had higher production rates than native SP variants. The mean producing antibody with IgE SP is 63 %, with its corresponding counterparts with native SPs at 22 %. The only exceptions where the native SPs had higher productions were the Vκ1|VH1, Vκ5|VH2, Vκ1|VH4 Vκ1|VH5, Vκ1|VH6 and Vκ1|VH7 pairs.

In the Pertuzumab dataset, the Vκ5 family is the sole poor producing family, whereas the low production families in the recombinant Trastuzumab model dataset (**Figure 2D**) extended to VH1, VH2, VH4 and VH6 (light chains that paired with these VHs had lower yields). One notable exception was the Vκ5|VH3 pair that was produced at higher levels compared to other Vκ5 family permutations in the Trastuzumab dataset (**Figure 2D**).

VH1 and VH7 genes shared the same SP (as classified in IMGT) amino acid sequence but with different codons. While these were normalized through codon optimization, there had distinct different productions between the two VH families, with VH7 being the better producing partner (averaged production of VH7 with its light chain partners at 48% when using native SPs and at 58 % when using IgE SP) compared to VH1 (at 37 % when using native SPs and at 48 % when using IgE SP) in the Pertuzumab dataset. The effect between VH1 and VH7 was even more pronounced in the Trastuzumab dataset, where VH1 recombinant production level were significantly reduced (at 7 % when using native SPs, and 20 % when using IgE SP) as compared to VH7 (at 37 % when using native SPs, and 110 % when using the IgE SP).

### Comparison of IgE, Vκ1 and Native SPs in Recombinant Pertuzumab and Trastuzumab Antibody Production

**Figure 3:**
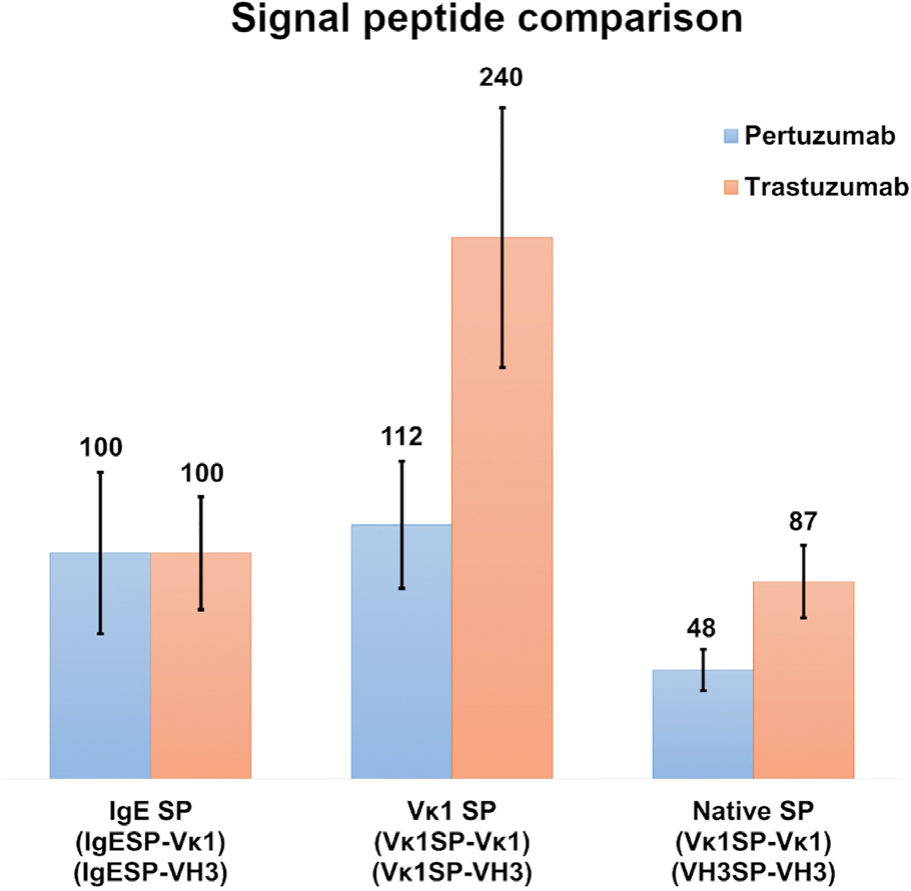
Recombinant antibody production rates of wild-type Vκ1|VH3 Pertuzumab (blue) and Trastuzumab (orange) using the IgE, Vκ1 and native SPs in %.

To investigate the role of light chain SPs, Vκ1 SP was grafted onto the Pertuzumab and Trastuzumab VH3 FWR and compared to the IgE and native SPs pairings after normalization with the respective Trastuzumab/Pertuzumab variants with the IgE SP (**Figure 3**). The respective heavy and light chains of Pertuzumab/Trastuzumab with the IgE SP are termed IgESP-Vκ1 (light chain) and IgESP-VH3 (heavy chain) in **Figure 3**. The native SPs (Vκ1SP-Vκ1, VH3SP-VH3) had the lowest recombinant production at 48 % and 87 % for the Pertuzumab and Trastuzumab models, respectively. The Vκ1 SP (Vκ1SP-Vκ1, Vκ1SP-VH3) had the highest production at 112 % and 240 % in the Pertuzumab and Trastuzumab models, respectively.

### Myeloma SPs Production Rates

**Figure 4:**
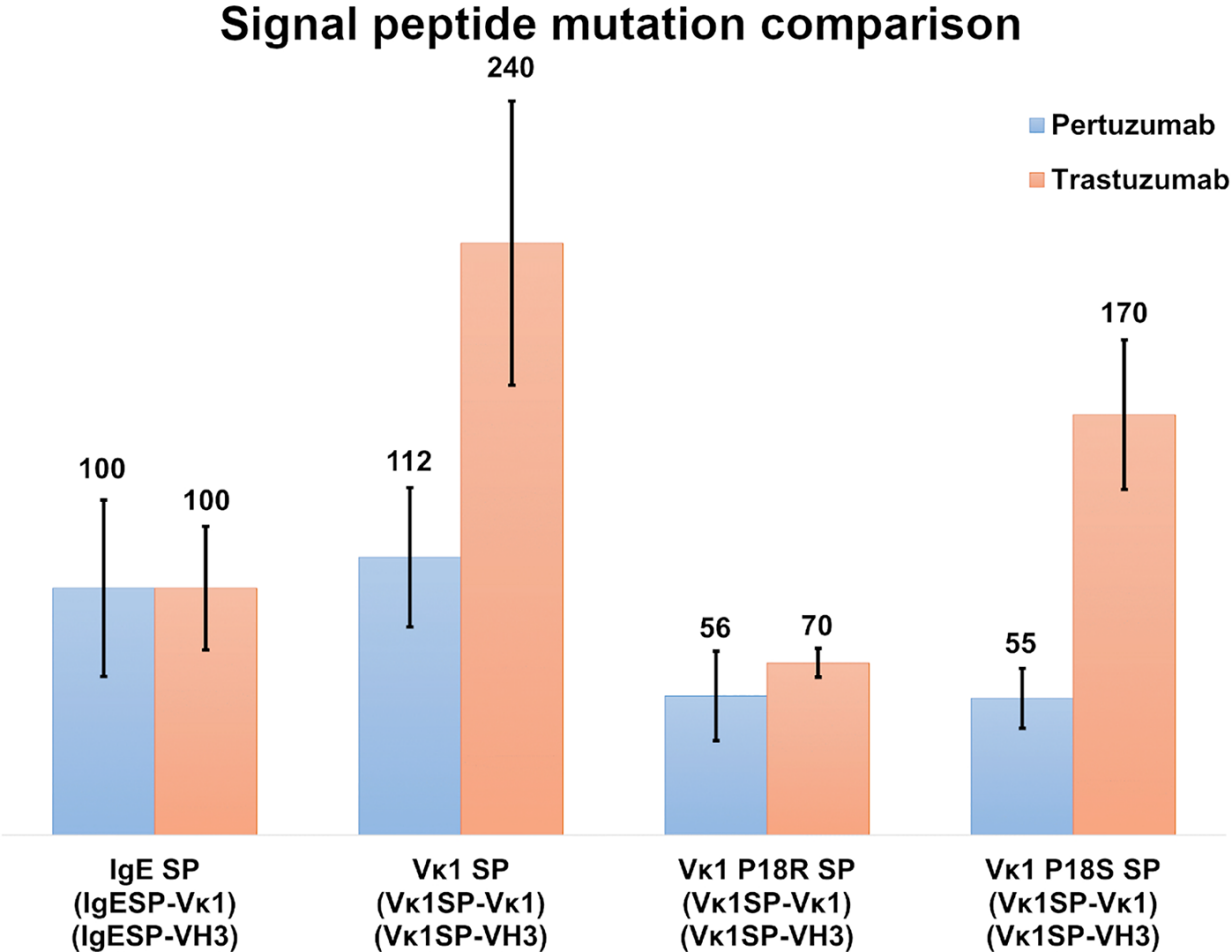
Recombinant antibody production (%) with IgE, Vκ1 and mutated Vκ1 SPs, (Vκ1 P18R and Vκ1 P18S) against the wild-type Vκ1|VH3 Pertuzumab (blue) and Trastuzumab (orange) models.

To study the effect of myeloma SPs on improving production levels, we performed single amino acid mutagenesis (P18R and P18S) on the Vκ1 SP (**Figure 4**). The mutated SPs were generated based on a previous reported myeloma Vκ1 SP associated with Fanconi’s syndrome SP (32), and the IgE SP sequence which was also from a myeloma patient (33). The Vκ1 P18R and P18S SPs showed 56 % & 55 % in Pertuzumab and 70 % & 170 % in Trastuzumab, respectively, compared to native Vκ1 SP at 112 % & 240 % in Pertuzumab and Trastuzumab, respectively. Both mutations had lower productions compared to the native Vκ1 SP, but the P18S SP Trastuzumab had notably higher recombinant production than the wild-type IgE SP.

### The Roles of Essential (EEA) and Non-essential (NEAA) Amino Acid on Antibody Production

With no clear correlation between production levels and the SP used, the content of the SPs was investigated. EAAs were found to make up half of the SP length (**Table 1**) with varied usage, where some amino acids were evidently more prominent, e.g. leucine (L).

**Table 1:** List of SP sequences with essential amino acids (EAAs, various colors) and non-essential amino acids (NEAAs, uncoloured) usage shown.

Family	Signal peptide amino acid position																						Total variety/No. of EAA
	1	2	3	4	5	6	7	8	9	10	11	12	13	14	15	16	17	18	19	20	21	22	
**Vκ1**	**M**	**D**	**M**	**R**	**V**	**P**	**A**	**Q**	**L**	**L**	**G**	**L**	**L**	**L**	**L**	**W**	**L**	**P**	**G**	**A**	**R**	**C**	**4/11**
**Vκ2**	**M**	**R**	**L**	**P**	**A**	**Q**	**L**	**L**	**G**	**L**	**L**	**M**	**L**	**W**	**V**	**P**	**G**	**S**	**S**	**A**			**4/10**
**Vκ3**	**M**	**E**	**A**	**P**	**A**	**Q**	**L**	**L**	**F**	**L**	**L**	**L**	**L**	**W**	**L**	**P**	**D**	**T**	**T**	**G**			**5/12**
**Vκ4**	**M**	**V**	**L**	**Q**	**T**	**Q**	**V**	**F**	**I**	**S**	**L**	**L**	**L**	**W**	**I**	**S**	**G**	**A**	**Y**	**G**			**7/12**
**Vκ5**	**M**	**G**	**S**	**Q**	**V**	**H**	**L**	**L**	**S**	**F**	**L**	**L**	**L**	**W**	**I**	**S**	**D**	**T**	**R**	**A**			**8/12**
**Vκ6**	**M**	**L**	**P**	**S**	**Q**	**L**	**I**	**G**	**F**	**L**	**L**	**L**	**W**	**V**	**P**	**A**	**S**	**R**	**G**				**6/10**
**VH1**	**M**	**D**	**W**	**T**	**W**	**R**	**I**	**L**	**F**	**L**	**V**	**A**	**A**	**A**	**T**	**G**	**A**	**H**	**S**				**8/11**
**VH2**	**M**	**D**	**I**	**L**	**C**	**S**	**T**	**L**	**L**	**L**	**L**	**T**	**V**	**P**	**S**	**W**	**V**	**L**	**S**				**6/13**
**VH3**	**M**	**E**	**F**	**G**	**L**	**S**	**W**	**V**	**F**	**L**	**V**	**A**	**I**	**L**	**K**	**G**	**V**	**Q**	**C**				**7/12**
**VH4**	**M**	**K**	**H**	**L**	**W**	**F**	**F**	**L**	**L**	**L**	**V**	**A**	**A**	**P**	**R**	**W**	**V**	**L**	**S**				**7/14**
**VH5**	**M**	**G**	**S**	**T**	**A**	**I**	**L**	**A**	**L**	**L**	**L**	**A**	**V**	**L**	**Q**	**G**	**V**	**C**	**S**				**5/10**
**VH6**	**M**	**S**	**V**	**S**	**F**	**L**	**I**	**F**	**L**	**P**	**V**	**L**	**G**	**L**	**P**	**W**	**G**	**V**	**L**	**S**			**6/13**
**VH7**	**M**	**D**	**W**	**T**	**W**	**R**	**I**	**L**	**F**	**L**	**V**	**A**	**A**	**A**	**T**	**G**	**A**	**H**	**S**				**8/11**
**IgE**	**M**	**D**	**W**	**T**	**W**	**I**	**L**	**F**	**L**	**V**	**A**	**A**	**A**	**T**	**R**	**V**	**H**	**S**					**8/12**

Comparisons of the total variety counts of EAAs in the SPs explored in this study.

Light chain SPs were found to be longer in length, more homogenous in amino acid type usage (average 5–6 variety EAA) with lower average EAA counts (11–12 amino acids) than the heavy chain SPs. The heavy chain SPs were generally shorter in length, more varied in amino acid types (average 7–8 variety EAA types) with higher average EAA counts (13–14 amino acids). This suggests that EAAs may be the underlying reason to why Vκ1 SP yielded better production rates than the antibodies using IgE SP (**Figures 2** and **3**), which in turn, had better production than those using the native SPs.

**Figure 5:**
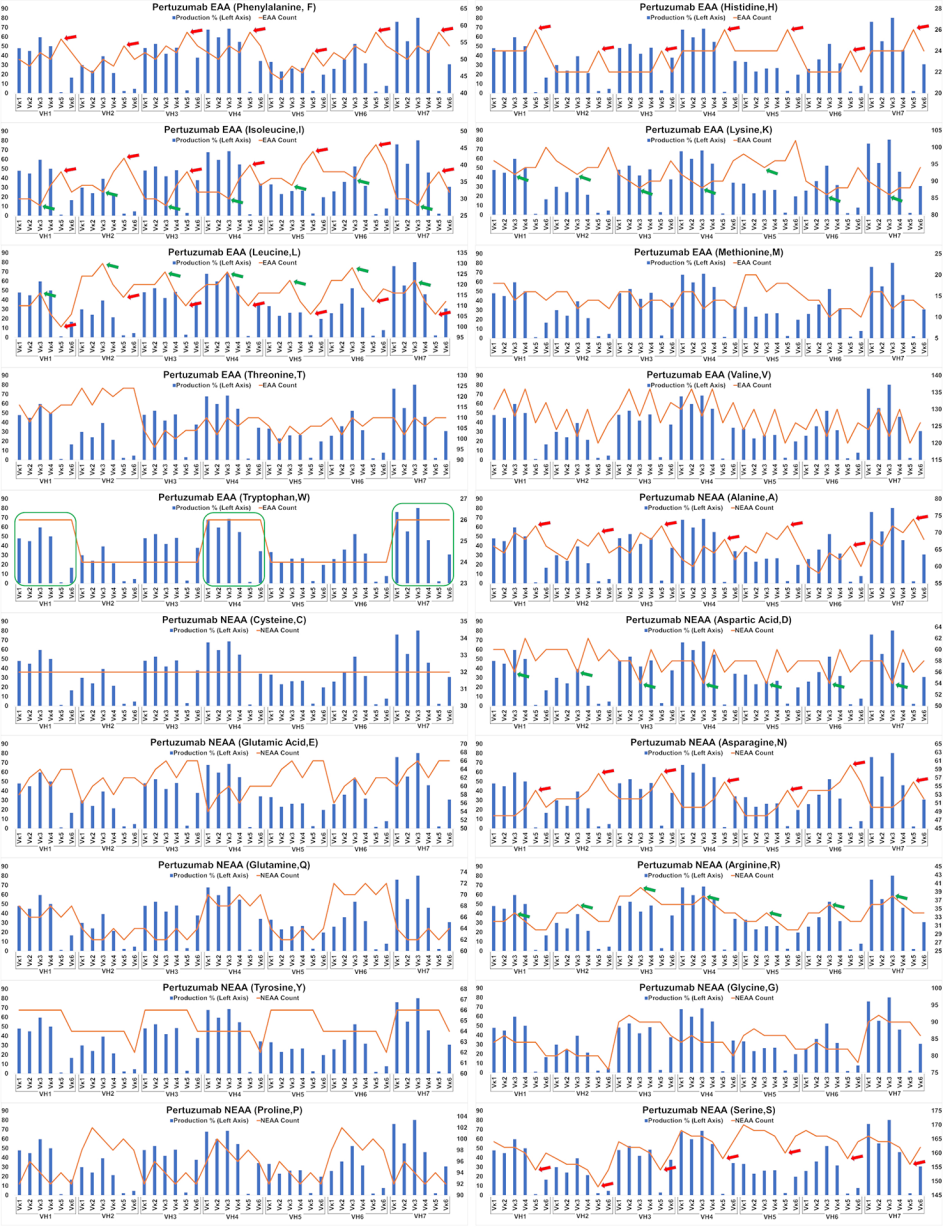
Production in % (in bars, left axis) and amino acid counts (line, right axis) charts of recombinant Pertuzumab variants. The green arrows/boxes refer to an increase in production level while the red arrows depict a decrease in production level.

The impact of EAA and NEAA usage would reasonably extend beyond just the SP to the whole protein. Applying this analysis to the full-length Pertuzumab and Trastuzumab variants (including light and heavy chains), we found the trend that higher counts of phenylalanine (F), histidine (H), isoleucine (I), alanine (A), asparagine, (N) and lower counts of leucine (L) and serine (S) within the Pertuzumab variants (**Figure 5**) may contribute to the poorer production levels of the Vκ5 family. We observed similar trends that higher counts of L, arginine (R) and lower counts of I, lysine (K), and aspartic acid (D) may account for the increased production in the Vκ3 family with higher counts of tryptophan (W) possibly accounting for higher production in general.

**Figure 6:**
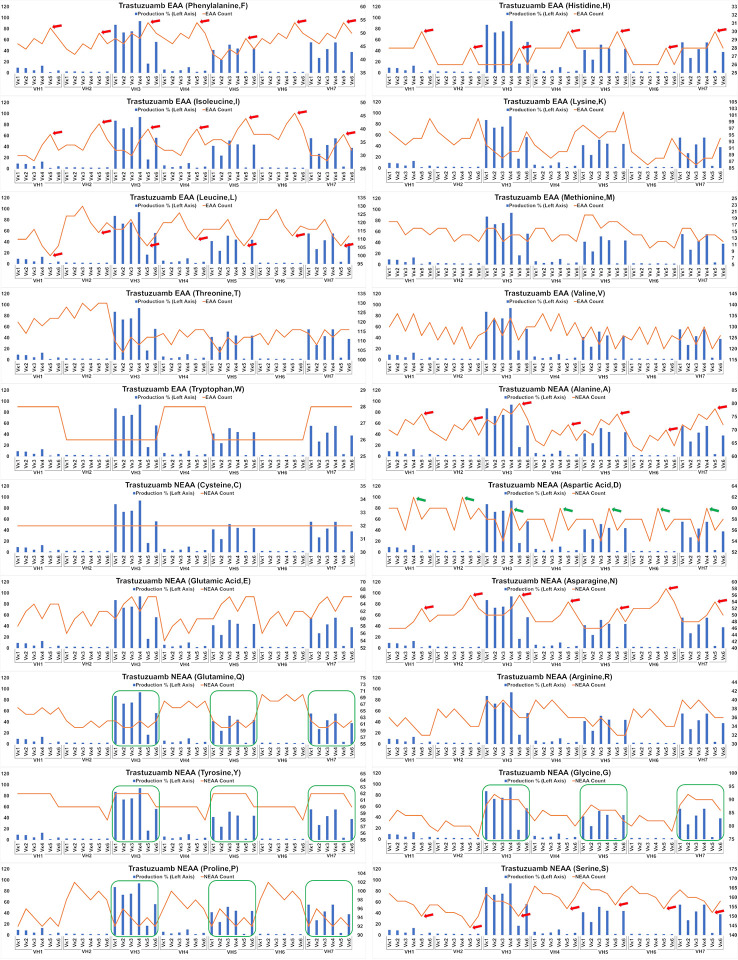
Production in % (in bars, left axis) and amino acid counts (line, right axis) charts of recombinant Trastuzumab variants. The green arrows/boxes refer to an increase in production level while the red arrows depict a decrease in production level.

Analysis of the Trastuzumab variants (**Figure 6**) showed similar trends in amino acids usage due to the high similarities of CDRs between Trastuzumab and Pertuzumab. This extended to the poor production in Vκ5 of both Trastuzumab (**Figure 6**) and Pertuzumab (**Figure 5**). Higher counts of D appear to improve production in Trastuzumab Vκ4 variants, contrary to Pertuzumab Vκ3 variants. Higher counts of Tyrosine (Y), Proline (P), Glycine (G) and lower counts of Glutamine (Q) appear to be associated with better production in our Trastuzumab repertoire.

### The Effects of Amino Acid Supply on Recombinant Antibody Production

Since the culture media is the predominant nutrient source in transient recombinant protein production, we analyzed the amino acid constituents and the demand for the respective amino acids (**Table 2**). For simpler analysis, we deemed batch variations involving co-transfection procedure variations and serum differences to be negligible. Based on the DMEM formulation (Sigma Aldrich, Cat no. D1152), W was found to be the limiting amino acid (0.47 × 10^20^ molecules) compared to other EAAs, restricting our maximum production to 1.88 × 10^18^ of antibodies (a unified representative average of amino acid usage from all the antibodies in this study, see **Supplementary Tables 1** and **2**).

**Table 2:** Amino acid counts in DMEM media and the representative average of amino acid usage in all the antibodies in this study.

		AA counts in full-length antibody	Number of AA in DMEM media (10^20^)	Maximum number of possible antibodies produced (10^18^)
**EAAs**	Phenylalanine (F)	50	2.41	4.82
	Histidine (H)	26	1.63	6.27
	Isoleucine (I)	34	4.82	14.18
	Lysine (K)	92	6.01	6.53
	Leucine (L)	116	4.82	4.16
	Methionine (M)	14	1.21	8.64
	Threonine (T)	112	4.80	4.29
	Valine (V)	128	4.83	3.77
	Tryptophan (W)	25	0.47	1.88
**NEAAs**	Alanine (A)	68	0	0
	Tyrosine (Y)	62	3.45	5.56
	Serine (S)	160	2.41	1.51
	Cysteine (C)	32	3.11	9.72
	Glycine (G)	84	2.41	2.87
	Aspartic Acid (D)	58	0	0
	Asparagine (N)	51	0	0
	Glutamic Acid (E)	62	0	0
	Proline (P)	96	0	0
	Glutamine(Q)	63	24.07	38.21
	Arginine (R)	35	2.91	8.31

The number of amino acids in DMEM media were calculated based on the weight (g/L) of amino acid component used in the formula. Maximum number of antibodies produced refers to the theoretical maximum number of full-length antibodies that could be synthesized based on number of amino acids in DMEM media and to the amino acid counts in the representative average of amino acids.

Five NEAAs: A, D, E, N and P were not provided for in DMEM. This is probably assumed by media makers to be sufficient from internal cell synthesis. However, three NEAAs: S, D, and Glutamic acid (E) are precursors to Cysteine (C), G, N, P, Q, and R but were also absent in the media, making these amino acids potential limiting factors.

**Figure 7:**
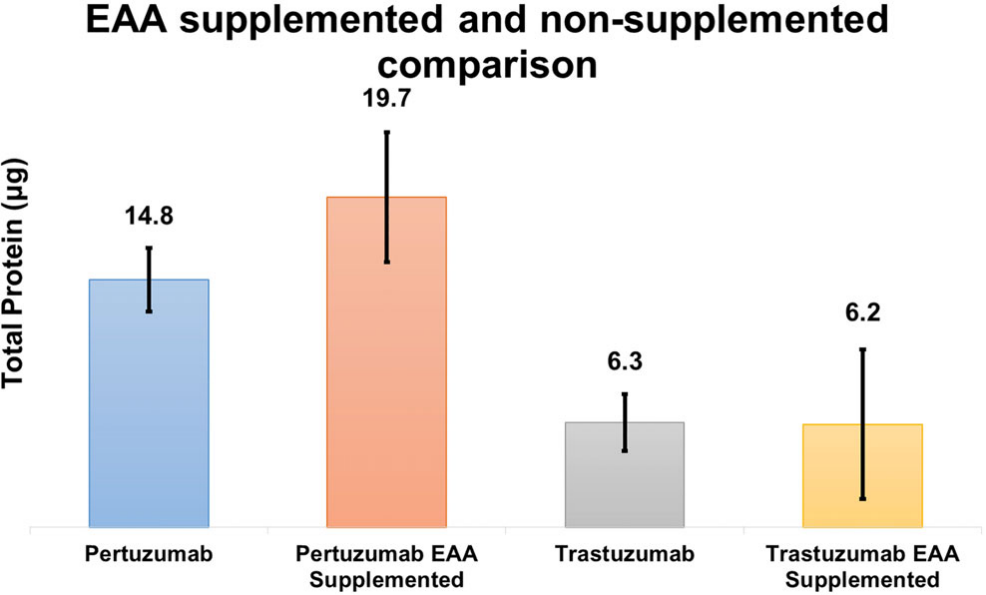
Quantification comparison of transient recombinant antibody production between EAA spiked and non-spiked for POK PG1 and HOK HG1 constructs.

Based on the theoretical assumption shown in **Table 2**, we performed a small scale transfection test (**Figure 7**) to determine if the addition of EAAs would improve the protein production. The results showed that Pertuzumab POK PG1 (IgE SP) had an average increment in total antibody production of 4.9 μg in 2 ml cultures when the media was supplemented with 7 mg of EAA compared to non-supplemented controls. While Trastuzumab HOK HG1 did not show significant average total antibody production differences when supplemented, two out of three independent replicates showed increases (**Supplementary Table 3**).

### A Statistical Predictive Model of Antibody Production Rate

Involvement of the amino acid counts were used to construct a statistical model to computationally predict the antibody production rates based on our co-transfection transient HEK293 cell system. Data from the current study and our previous work (24) were used.

**Figure 8:**
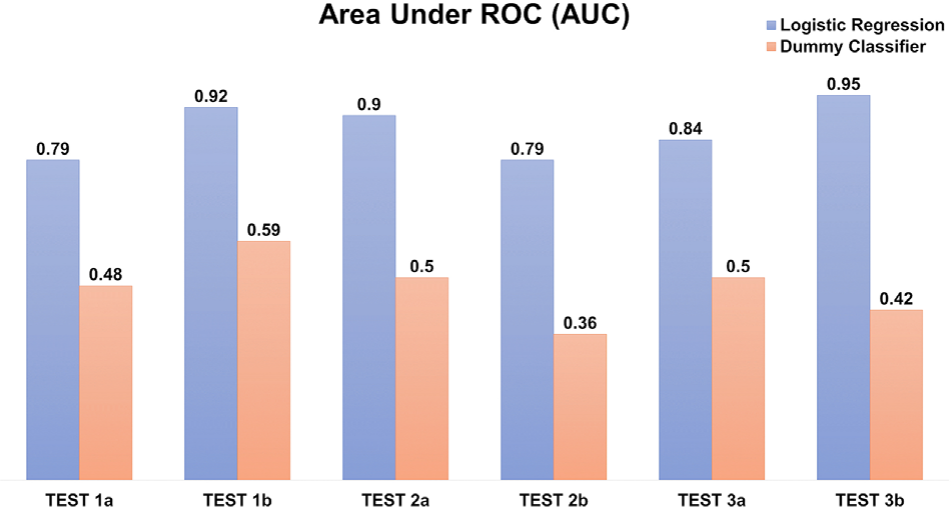
Logistic regression model to predict the recombinant antibody production rates using amino acid counts. The prediction is evaluated using Area under the ROC (AUC) in triplicates, each with two independents **(A, B)** testing datasets. The “dummy” classifier is used as a baseline control.

The prediction scores on the testing datasets of AUC ~0.79–0.95 (**Figure 8**) and F1-score 0.62–0.7 (of which 1 reflecting the best balance between precision and recall), depicted a reasonable prediction model for production rate categories (low, medium, or high).

## Discussion

We investigated the effects of numerous antibody signal peptides (SP) on recombinant antibody production in a co-transfection transient system using HEK293 cells, normalizing with the same transfection agents and backbone plasmids to varying only the signal peptides. We found that the IgE SP generally gave better yields than the native SPs (**Figure 2**).

To study possible effects of SPs from heavy and light chains (13), we grafted the Vκ1 SP on the wild-type Vκ1|VH3 of the recombinant Pertuzumab and Trastuzumab (**Figure 3**) for comparison to IgE SP and the respective native SP counterparts. Both IgE and Vκ1 SPs yielded better productions. With the possibility that myeloma SP might give better production, we studied another myeloma-linked SP – a variant of Vκ1 SP -with P18R or P18S different from the Vκ1 SP in IMGT (32).

Grafting of the myeloma Vκ1 SP onto the wild-type Vκ1|VH3 recombinant Pertuzumab and Trastuzumab models showed that mutation P18S increased production in the Trastuzumab model but resulted in decreased production in the Pertuzumab model, while mutation P18R resulted in decreased production in both Trastuzumab and Pertuzumab models, compared to the native Vκ1 SP (**Figure 4**). Given these myeloma SP mutation findings, we did not find support for the role of SP in hyperglobulinemia pathogenesis within myeloma, and that there remains an enigmatic relationship between the SP and antibody production.

Analyzing the nine essential amino acid (EAA) content in SP sequences, especially given that previous experiments showed that the addition of supplements may be helpful in protein production, we found light chain SPs to have a lower variety and number of EAAs usage than the heavy chain SPs (**Table 1**), providing a clue to their possible contribution to recombinant production.

Extending the essential and non-essential amino acid analysis (**Figures 5** and **6**) to both full-length Pertuzumab and Trastuzumab variants, we found that the presence of F, H, I, A, N, L and S amino acids may underlie the poor production of Vκ5 Pertuzumab and Trastuzumab variants. On the other hand, L, R, I, K and D amino acids may allow for better production as seen with specific variants paired with Vκ3 (Pertuzumab) and Vκ4 (Trastuzumab). Within these trends, there were certain exceptions such as Pertuzumab Vκ6 that despite having similar counts of respective amino acid (e.g. F and H), did not have comparable production levels to better producing families such as VK1-4l or Trastuzumab VH2, both of which had having similar count of Q as with high producing VH3, 5 & 7 but it resulted in low production.

DMEM formulation (**Table 2**) provided insufficient amino acids to support optimal antibodies production, especially since EEA: W, and NEAAs: A, D, E, N, and P, were not provided at all nor present in inadequate quantities. This thus explained the boost in production when supplements such as peptone and casein were added (34–37). In our follow up experiment (**Figure 7**), we also found EAA supplements to increase overall antibody production in both the Pertuzumab and Trastuzumab models, thereby providing a possible supplement for academic and industrial antibody production.

Our predictive model based on amino acid accounts (**Figure 8**) was able to determine the ordinal production levels (low, medium, or high) with high accuracy (ROC AUC ~0.79–0.95). Nonetheless, the model is sensitive to the imbalanced datasets (e.g. 15–20 % of the “high” producers) despite optimizing using weighted parameters. In addition, it is confined by the transient expression conditions used in our experiments. Nonetheless, other similar transfection data can be incorporated to further improve the model for wider applicability in future work.

With amino acids count being a factor in antibody production rates, the underlying reason that antibody genes utilize such a big repertoire of SPs may be rationalized. It is possible that the repertoire may serve to mitigate over reliance of specific EAAs that impact antibody production. Considering that the hypervariable antibody VDJ genes (38) utilize a wide permutation of the amino acids, a fixed signal peptide for all antibodies can increase the probability of heavy bias towards specific amino acids. Such biases can thus create bottlenecks to certain EAA(s) and hamper not only antibody production, but also essential cellular protein production. Therefore, by varying the SPs with differing EAA content, such bottlenecks of EAAs may be mitigated, thus explaining a possible function of the large repertoire of SPs in antibody genes.

In conclusion, the study of antibodies SP with EAA factors provides a new approach on the understanding of transient mammalian protein production and possible insights to the repertoire of SPs utilized by antibodies. These new EAA factors could have potential for wide-spread application to other production systems in a better understanding of protein production.

## Data Availability Statement

The raw data supporting the conclusions of this article will be made available by the authors, without undue reservation.

## Author Contributions

Conceptualization, W-LL and SK-EG; Methodology, W-LL; Investigation, W-LL and CT-TS; Validation, W-HL and J-JP; Writing – Original Draft, W-LL and CT-TS; Writing – Review & Editing, W-LL, CT-TS, Y-LN and SK-EG; Funding Acquisition, SK-EG; Supervision, SK-EG, Y-LN and AW.

## Funding

This work was supported by the Interstellar Initiative 2018-2019 jointly supported by the Japan Agency for Medical Research and Development and New York Academy of Sciences, and also, A*STAR.

## Conflict of Interest

The authors declare that the research was conducted in the absence of any commercial or financial relationships that could be construed as a potential conflict of interest.
