# Supplementary material for: Essentially Leading Antibody Production: An Investigation of Amino Acids, Myeloma, and Natural V-Region Signal Peptides in Producing Pertuzumab and Trastuzumab Variants
Source: Front Immunol. 2020 Dec 7;11:604318. doi: 10.3389/fimmu.2020.604318 (PMC7750424; doi:10.3389/fimmu.2020.604318)
Supplement: Supplementary file 1 [file DataSheet_1.docx]

Supplementary Material

# Supplementary Figures


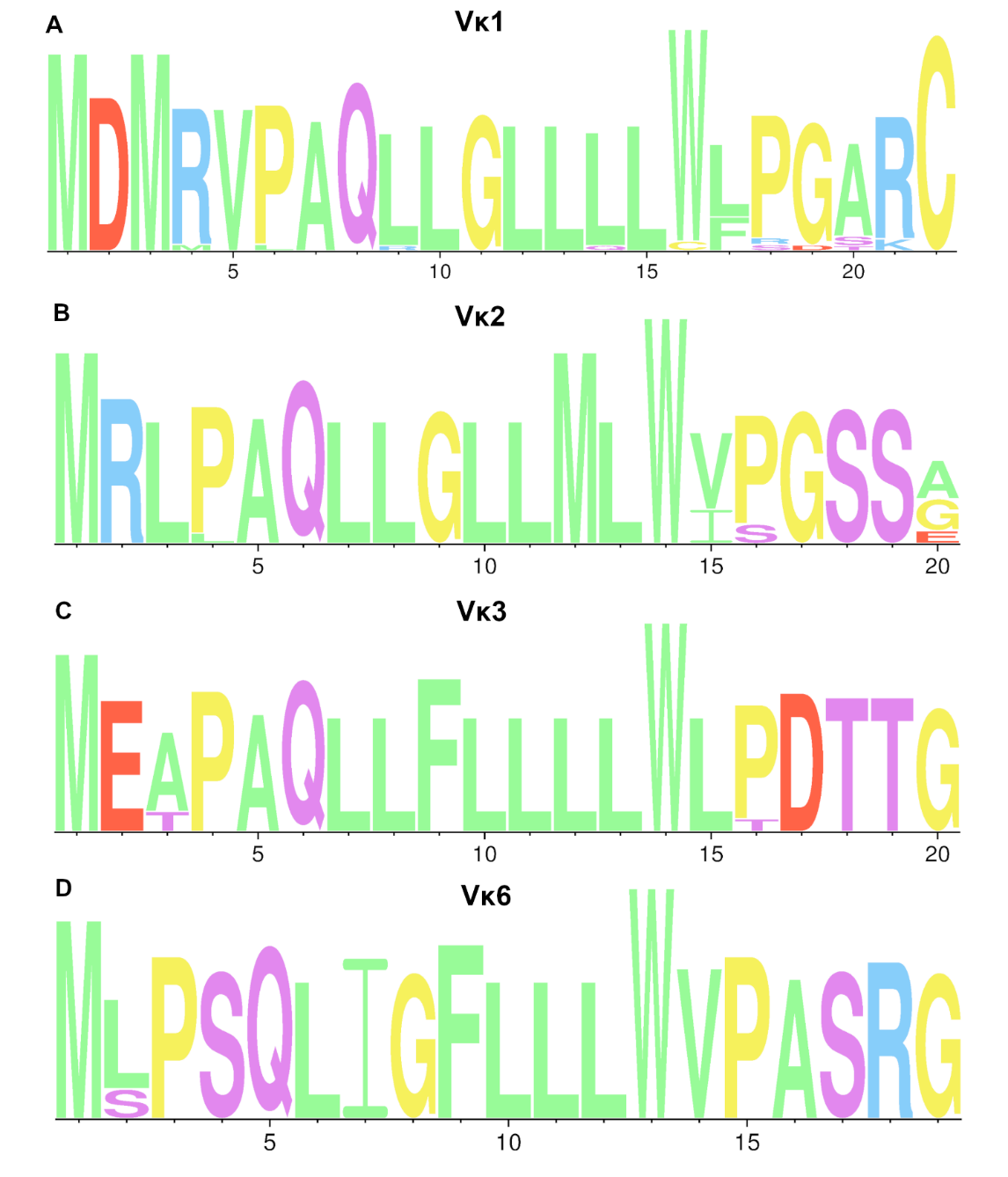


**Supplementary Figure 1.** Consensus sequence analysis of Vκ amino acid sequences using WebLogo (Crooks et al., 2004). Only Vκ1-3 and Vκ6 were shown as both Vκ4 and Vκ5 lacked sufficient sequences for consensus alignment. Blue, Red, Purple, Yellow and Green colors code for the amino acids with positive, negative charges, polar uncharged, special, and hydrophobic side chains, respectively.


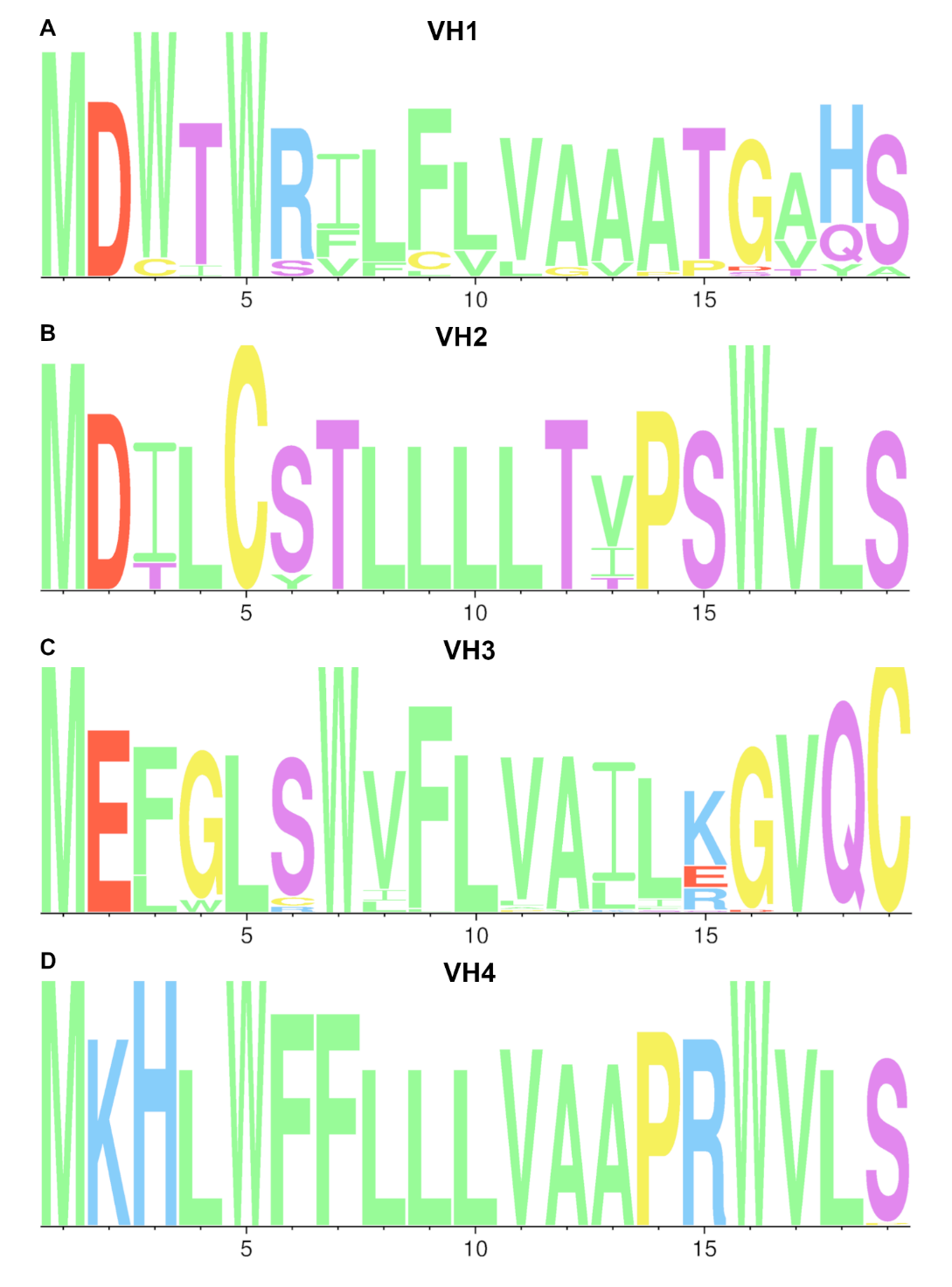


**Supplementary Figure 2.** Consensus sequence analysis of VH amino acid sequences using WebLogo (Crooks et al., 2004). Only VH1-4 were represented here as VH5-7 lacked sufficient sequences for consensus alignment. Blue, Red, Purple, Yellow and Green colors code for the amino acids with positive, negative charges, polar uncharged, special, and hydrophobic side chains, respectively

# Supplementary Tables

**Supplementary Table 1.** Full amino acid counts of Pertuzumab variants

| Pertuzumab | Amino acid | | | | | | | | | | | | | | | | | | | |
| --- | --- | --- | --- | --- | --- | --- | --- | --- | --- | --- | --- | --- | --- | --- | --- | --- | --- | --- | --- | --- |
|  | F | H | I | K | L | M | T | V | W | A | C | D | E | G | N | P | Q | R | S | Y |
| Vκ1\|VH1 | 50 | 24 | 30 | 96 | 110 | 18 | 116 | 130 | 26 | 66 | 32 | 60 | 58 | 84 | 48 | 92 | 68 | 32 | 164 | 66 |
| Vκ2\|VH1 | 48 | 24 | 30 | 94 | 110 | 18 | 108 | 136 | 26 | 64 | 32 | 60 | 62 | 86 | 48 | 96 | 66 | 32 | 162 | 66 |
| Vκ3\|VH1 | 52 | 24 | 28 | 92 | 116 | 14 | 116 | 128 | 26 | 70 | 32 | 56 | 64 | 84 | 48 | 94 | 66 | 34 | 162 | 66 |
| Vκ4\|VH1 | 50 | 24 | 34 | 94 | 106 | 16 | 112 | 136 | 26 | 68 | 32 | 62 | 60 | 84 | 50 | 92 | 68 | 32 | 160 | 66 |
| Vκ5\|VH1 | 56 | 26 | 38 | 94 | 100 | 16 | 116 | 126 | 26 | 72 | 32 | 58 | 64 | 84 | 54 | 94 | 66 | 30 | 154 | 66 |
| Vκ6\|VH1 | 52 | 24 | 32 | 100 | 106 | 14 | 116 | 132 | 26 | 66 | 32 | 60 | 64 | 80 | 50 | 92 | 68 | 30 | 160 | 64 |
| Vκ1\|VH2 | 48 | 22 | 34 | 96 | 124 | 16 | 124 | 124 | 24 | 64 | 32 | 60 | 56 | 80 | 52 | 98 | 64 | 34 | 158 | 64 |
| Vκ2\|VH2 | 46 | 22 | 34 | 94 | 124 | 16 | 116 | 130 | 24 | 62 | 32 | 60 | 60 | 82 | 52 | 102 | 62 | 34 | 156 | 64 |
| Vκ3\|VH2 | 50 | 22 | 32 | 92 | 130 | 12 | 124 | 122 | 24 | 68 | 32 | 56 | 62 | 80 | 52 | 100 | 62 | 36 | 156 | 64 |
| Vκ4\|VH2 | 48 | 22 | 38 | 94 | 120 | 14 | 120 | 130 | 24 | 66 | 32 | 62 | 58 | 80 | 54 | 98 | 64 | 34 | 154 | 64 |
| Vκ5\|VH2 | 54 | 24 | 42 | 94 | 114 | 14 | 124 | 120 | 24 | 70 | 32 | 58 | 62 | 80 | 58 | 100 | 62 | 32 | 148 | 64 |
| Vκ6\|VH2 | 50 | 22 | 36 | 100 | 120 | 12 | 124 | 126 | 24 | 64 | 32 | 60 | 62 | 76 | 54 | 98 | 64 | 32 | 154 | 62 |
| Vκ1\|VH3 | 52 | 22 | 30 | 92 | 120 | 16 | 104 | 130 | 24 | 66 | 32 | 58 | 60 | 90 | 52 | 92 | 64 | 38 | 164 | 66 |
| Vκ2\|VH3 | 50 | 22 | 30 | 90 | 120 | 16 | 96 | 136 | 24 | 64 | 32 | 58 | 64 | 92 | 52 | 96 | 62 | 38 | 162 | 66 |
| Vκ3\|VH3 | 54 | 22 | 28 | 88 | 126 | 12 | 104 | 128 | 24 | 70 | 32 | 54 | 66 | 90 | 52 | 94 | 62 | 40 | 162 | 66 |
| Vκ4\|VH3 | 52 | 22 | 34 | 90 | 116 | 14 | 100 | 136 | 24 | 68 | 32 | 60 | 62 | 90 | 54 | 92 | 64 | 38 | 160 | 66 |
| Vκ5\|VH3 | 58 | 24 | 38 | 90 | 110 | 14 | 104 | 126 | 24 | 72 | 32 | 56 | 66 | 90 | 58 | 94 | 62 | 36 | 154 | 66 |
| Vκ6\|VH3 | 54 | 22 | 32 | 96 | 116 | 12 | 104 | 132 | 24 | 66 | 32 | 58 | 66 | 86 | 54 | 92 | 64 | 36 | 160 | 64 |
| Vκ1\|VH4 | 52 | 24 | 32 | 92 | 120 | 16 | 110 | 130 | 26 | 62 | 32 | 58 | 54 | 84 | 50 | 96 | 70 | 36 | 168 | 64 |
| Vκ2\|VH4 | 50 | 24 | 32 | 90 | 120 | 16 | 102 | 136 | 26 | 60 | 32 | 58 | 58 | 86 | 50 | 100 | 68 | 36 | 166 | 64 |
| Vκ3\|VH4 | 54 | 24 | 30 | 88 | 126 | 12 | 110 | 128 | 26 | 66 | 32 | 54 | 60 | 84 | 50 | 98 | 68 | 38 | 166 | 64 |
| Vκ4\|VH4 | 52 | 24 | 36 | 90 | 116 | 14 | 106 | 136 | 26 | 64 | 32 | 60 | 56 | 84 | 52 | 96 | 70 | 36 | 164 | 64 |
| Vκ5\|VH4 | 58 | 26 | 40 | 90 | 110 | 14 | 110 | 126 | 26 | 68 | 32 | 56 | 60 | 84 | 56 | 98 | 68 | 34 | 158 | 64 |
| Vκ6\|VH4 | 54 | 24 | 34 | 96 | 116 | 12 | 110 | 132 | 26 | 62 | 32 | 58 | 60 | 80 | 52 | 96 | 70 | 34 | 164 | 62 |
| Vκ1\|VH5 | 46 | 24 | 36 | 98 | 116 | 20 | 106 | 124 | 24 | 66 | 32 | 58 | 60 | 86 | 48 | 92 | 64 | 32 | 170 | 66 |
| Vκ2\|VH5 | 44 | 24 | 36 | 96 | 116 | 20 | 98 | 130 | 24 | 64 | 32 | 58 | 64 | 88 | 48 | 96 | 62 | 32 | 168 | 66 |
| Vκ3\|VH5 | 48 | 24 | 34 | 94 | 122 | 16 | 106 | 122 | 24 | 70 | 32 | 54 | 66 | 86 | 48 | 94 | 62 | 34 | 168 | 66 |
| Vκ4\|VH5 | 46 | 24 | 40 | 96 | 112 | 18 | 102 | 130 | 24 | 68 | 32 | 60 | 62 | 86 | 50 | 92 | 64 | 32 | 166 | 66 |
| Vκ5\|VH5 | 52 | 26 | 44 | 96 | 106 | 18 | 106 | 120 | 24 | 72 | 32 | 56 | 66 | 86 | 54 | 94 | 62 | 30 | 160 | 66 |
| Vκ6\|VH5 | 48 | 24 | 38 | 102 | 112 | 16 | 106 | 126 | 24 | 66 | 32 | 58 | 66 | 82 | 50 | 92 | 64 | 30 | 166 | 64 |
| Vκ1\|VH6 | 52 | 22 | 38 | 90 | 122 | 14 | 110 | 124 | 24 | 60 | 32 | 58 | 56 | 82 | 54 | 98 | 72 | 34 | 168 | 64 |
| Vκ2\|VH6 | 50 | 22 | 38 | 88 | 122 | 14 | 102 | 130 | 24 | 58 | 32 | 58 | 60 | 84 | 54 | 102 | 70 | 34 | 166 | 64 |
| Vκ3\|VH6 | 54 | 22 | 36 | 86 | 128 | 10 | 110 | 122 | 24 | 64 | 32 | 54 | 62 | 82 | 54 | 100 | 70 | 36 | 166 | 64 |
| Vκ4\|VH6 | 52 | 22 | 42 | 88 | 118 | 12 | 106 | 130 | 24 | 62 | 32 | 60 | 58 | 82 | 56 | 98 | 72 | 34 | 164 | 64 |
| Vκ5\|VH6 | 58 | 24 | 46 | 88 | 112 | 12 | 110 | 120 | 24 | 66 | 32 | 56 | 62 | 82 | 60 | 100 | 70 | 32 | 158 | 64 |
| Vκ6\|VH6 | 54 | 22 | 40 | 94 | 118 | 10 | 110 | 126 | 24 | 60 | 32 | 58 | 62 | 78 | 56 | 98 | 72 | 32 | 164 | 62 |
| Vκ1\|VH7 | 52 | 24 | 30 | 90 | 116 | 16 | 110 | 124 | 26 | 68 | 32 | 58 | 60 | 90 | 50 | 92 | 64 | 36 | 166 | 66 |
| Vκ2\|VH7 | 50 | 24 | 30 | 88 | 116 | 16 | 102 | 130 | 26 | 66 | 32 | 58 | 64 | 92 | 50 | 96 | 62 | 36 | 164 | 66 |
| Vκ3\|VH7 | 54 | 24 | 28 | 86 | 122 | 12 | 110 | 122 | 26 | 72 | 32 | 54 | 66 | 90 | 50 | 94 | 62 | 38 | 164 | 66 |
| Vκ4\|VH7 | 52 | 24 | 34 | 88 | 112 | 14 | 106 | 130 | 26 | 70 | 32 | 60 | 62 | 90 | 52 | 92 | 64 | 36 | 162 | 66 |
| Vκ5\|VH7 | 58 | 26 | 38 | 88 | 106 | 14 | 110 | 120 | 26 | 74 | 32 | 56 | 66 | 90 | 56 | 94 | 62 | 34 | 156 | 66 |
| Vκ6\|VH7 | 54 | 24 | 32 | 94 | 112 | 12 | 110 | 126 | 26 | 68 | 32 | 58 | 66 | 86 | 52 | 92 | 64 | 34 | 162 | 64 |
| **MEDIAN** | **52** | **24** | **34** | **92** | **116** | **14** | **110** | **128** | **24** | **66** | **32** | **58** | **62** | **84** | **52** | **96** | **64** | **34** | **162** | **64** |

**Supplementary Table 2.** Full amino acid counts of Trastuzumab variants

| Trastuzumab | Amino acid | | | | | | | | | | | | | | | | | | | |
| --- | --- | --- | --- | --- | --- | --- | --- | --- | --- | --- | --- | --- | --- | --- | --- | --- | --- | --- | --- | --- |
|  | F | H | I | K | L | M | T | V | W | A | C | D | E | G | N | P | Q | R | S | Y |
| Vκ1\|VH1 | 46 | 28 | 30 | 96 | 110 | 18 | 120 | 130 | 28 | 70 | 32 | 60 | 58 | 82 | 46 | 92 | 66 | 36 | 162 | 62 |
| Vκ2\|VH1 | 44 | 28 | 30 | 94 | 110 | 18 | 114 | 136 | 28 | 68 | 32 | 60 | 62 | 86 | 46 | 96 | 64 | 34 | 158 | 62 |
| Vκ3\|VH1 | 48 | 28 | 28 | 92 | 116 | 14 | 122 | 128 | 28 | 74 | 32 | 56 | 64 | 84 | 46 | 94 | 64 | 36 | 158 | 62 |
| Vκ4\|VH1 | 46 | 28 | 34 | 94 | 106 | 16 | 118 | 136 | 28 | 72 | 32 | 62 | 60 | 84 | 48 | 92 | 66 | 34 | 156 | 62 |
| Vκ5\|VH1 | 52 | 30 | 38 | 94 | 100 | 16 | 122 | 126 | 28 | 76 | 32 | 58 | 64 | 84 | 52 | 94 | 64 | 32 | 150 | 62 |
| Vκ6\|VH1 | 48 | 28 | 32 | 100 | 106 | 14 | 122 | 132 | 28 | 70 | 32 | 60 | 64 | 80 | 48 | 92 | 66 | 32 | 156 | 60 |
| Vκ1\|VH2 | 44 | 26 | 34 | 96 | 124 | 16 | 128 | 124 | 26 | 68 | 32 | 60 | 56 | 78 | 50 | 98 | 62 | 38 | 156 | 60 |
| Vκ2\|VH2 | 42 | 26 | 34 | 94 | 124 | 16 | 122 | 130 | 26 | 66 | 32 | 60 | 60 | 82 | 50 | 102 | 60 | 36 | 152 | 60 |
| Vκ3\|VH2 | 46 | 26 | 32 | 92 | 130 | 12 | 130 | 122 | 26 | 72 | 32 | 56 | 62 | 80 | 50 | 100 | 60 | 38 | 152 | 60 |
| Vκ4\|VH2 | 44 | 26 | 38 | 94 | 120 | 14 | 126 | 130 | 26 | 70 | 32 | 62 | 58 | 80 | 52 | 98 | 62 | 36 | 150 | 60 |
| Vκ5\|VH2 | 50 | 28 | 42 | 94 | 114 | 14 | 130 | 120 | 26 | 74 | 32 | 58 | 62 | 80 | 56 | 100 | 60 | 34 | 144 | 60 |
| Vκ6\|VH2 | 46 | 26 | 36 | 100 | 120 | 12 | 130 | 126 | 26 | 68 | 32 | 60 | 62 | 76 | 52 | 98 | 62 | 34 | 150 | 58 |
| Vκ1\|VH3 | 48 | 26 | 32 | 92 | 116 | 16 | 110 | 128 | 26 | 74 | 32 | 58 | 60 | 88 | 50 | 92 | 62 | 40 | 162 | 62 |
| Vκ2\|VH3 | 46 | 26 | 32 | 90 | 116 | 16 | 104 | 134 | 26 | 72 | 32 | 58 | 64 | 92 | 50 | 96 | 60 | 38 | 158 | 62 |
| Vκ3\|VH3 | 50 | 26 | 30 | 88 | 122 | 12 | 112 | 126 | 26 | 78 | 32 | 54 | 66 | 90 | 50 | 94 | 60 | 40 | 158 | 62 |
| Vκ4\|VH3 | 48 | 26 | 36 | 90 | 112 | 14 | 108 | 134 | 26 | 76 | 32 | 60 | 62 | 90 | 52 | 92 | 62 | 38 | 156 | 62 |
| Vκ5\|VH3 | 54 | 28 | 40 | 90 | 106 | 14 | 112 | 124 | 26 | 80 | 32 | 56 | 66 | 90 | 56 | 94 | 60 | 36 | 150 | 62 |
| Vκ6\|VH3 | 50 | 26 | 34 | 96 | 112 | 12 | 112 | 130 | 26 | 74 | 32 | 58 | 66 | 86 | 52 | 92 | 62 | 36 | 156 | 60 |
| Vκ1\|VH4 | 48 | 28 | 32 | 92 | 120 | 16 | 114 | 130 | 28 | 66 | 32 | 58 | 54 | 82 | 48 | 96 | 68 | 40 | 166 | 60 |
| Vκ2\|VH4 | 46 | 28 | 32 | 90 | 120 | 16 | 108 | 136 | 28 | 64 | 32 | 58 | 58 | 86 | 48 | 100 | 66 | 38 | 162 | 60 |
| Vκ3\|VH4 | 50 | 28 | 30 | 88 | 126 | 12 | 116 | 128 | 28 | 70 | 32 | 54 | 60 | 84 | 48 | 98 | 66 | 40 | 162 | 60 |
| Vκ4\|VH4 | 48 | 28 | 36 | 90 | 116 | 14 | 112 | 136 | 28 | 68 | 32 | 60 | 56 | 84 | 50 | 96 | 68 | 38 | 160 | 60 |
| Vκ5\|VH4 | 54 | 30 | 40 | 90 | 110 | 14 | 116 | 126 | 28 | 72 | 32 | 56 | 60 | 84 | 54 | 98 | 66 | 36 | 154 | 60 |
| Vκ6\|VH4 | 50 | 28 | 34 | 96 | 116 | 12 | 116 | 132 | 28 | 66 | 32 | 58 | 60 | 80 | 50 | 96 | 68 | 36 | 160 | 58 |
| Vκ1\|VH5 | 42 | 28 | 36 | 98 | 116 | 20 | 110 | 124 | 26 | 70 | 32 | 58 | 60 | 84 | 46 | 92 | 62 | 36 | 168 | 62 |
| Vκ2\|VH5 | 40 | 28 | 36 | 96 | 116 | 20 | 104 | 130 | 26 | 68 | 32 | 58 | 64 | 88 | 46 | 96 | 60 | 34 | 164 | 62 |
| Vκ3\|VH5 | 44 | 28 | 34 | 94 | 122 | 16 | 112 | 122 | 26 | 74 | 32 | 54 | 66 | 86 | 46 | 94 | 60 | 36 | 164 | 62 |
| Vκ4\|VH5 | 42 | 28 | 40 | 96 | 112 | 18 | 108 | 130 | 26 | 72 | 32 | 60 | 62 | 86 | 48 | 92 | 62 | 34 | 162 | 62 |
| Vκ5\|VH5 | 48 | 30 | 44 | 96 | 106 | 18 | 112 | 120 | 26 | 76 | 32 | 56 | 66 | 86 | 52 | 94 | 60 | 32 | 156 | 62 |
| Vκ6\|VH5 | 44 | 28 | 38 | 102 | 112 | 16 | 112 | 126 | 26 | 70 | 32 | 58 | 66 | 82 | 48 | 92 | 62 | 32 | 162 | 60 |
| Vκ1\|VH6 | 48 | 26 | 38 | 90 | 122 | 14 | 114 | 124 | 26 | 64 | 32 | 58 | 56 | 80 | 52 | 98 | 70 | 38 | 166 | 60 |
| Vκ2\|VH6 | 46 | 26 | 38 | 88 | 122 | 14 | 108 | 130 | 26 | 62 | 32 | 58 | 60 | 84 | 52 | 102 | 68 | 36 | 162 | 60 |
| Vκ3\|VH6 | 50 | 26 | 36 | 86 | 128 | 10 | 116 | 122 | 26 | 68 | 32 | 54 | 62 | 82 | 52 | 100 | 68 | 38 | 162 | 60 |
| Vκ4\|VH6 | 48 | 26 | 42 | 88 | 118 | 12 | 112 | 130 | 26 | 66 | 32 | 60 | 58 | 82 | 54 | 98 | 70 | 36 | 160 | 60 |
| Vκ5\|VH6 | 54 | 28 | 46 | 88 | 112 | 12 | 116 | 120 | 26 | 70 | 32 | 56 | 62 | 82 | 58 | 100 | 68 | 34 | 154 | 60 |
| Vκ6\|VH6 | 50 | 26 | 40 | 94 | 118 | 10 | 116 | 126 | 26 | 64 | 32 | 58 | 62 | 78 | 54 | 98 | 70 | 34 | 160 | 58 |
| Vκ1\|VH7 | 48 | 28 | 30 | 90 | 116 | 16 | 114 | 124 | 28 | 72 | 32 | 58 | 60 | 88 | 48 | 92 | 62 | 40 | 164 | 62 |
| Vκ2\|VH7 | 46 | 28 | 30 | 88 | 116 | 16 | 108 | 130 | 28 | 70 | 32 | 58 | 64 | 92 | 48 | 96 | 60 | 38 | 160 | 62 |
| Vκ3\|VH7 | 50 | 28 | 28 | 86 | 122 | 12 | 116 | 122 | 28 | 76 | 32 | 54 | 66 | 90 | 48 | 94 | 60 | 40 | 160 | 62 |
| Vκ4\|VH7 | 48 | 28 | 34 | 88 | 112 | 14 | 112 | 130 | 28 | 74 | 32 | 60 | 62 | 90 | 50 | 92 | 62 | 38 | 158 | 62 |
| Vκ5\|VH7 | 54 | 30 | 38 | 88 | 106 | 14 | 116 | 120 | 28 | 78 | 32 | 56 | 66 | 90 | 54 | 94 | 60 | 36 | 152 | 62 |
| Vκ6\|VH7 | 50 | 28 | 32 | 94 | 112 | 12 | 116 | 126 | 28 | 72 | 32 | 58 | 66 | 86 | 50 | 92 | 62 | 36 | 158 | 60 |
| **MEDIAN** | **48** | **28** | **34** | **92** | **116** | **14** | **114** | **128** | **26** | **70** | **32** | **58** | **62** | **84** | **50** | **96** | **62** | **36** | **158** | **60** |

**Supplementary Table 3.** Technical replicates of EEA supplemented and non-supplemented production of Pertuzumab POK PG1 and Trastuzumab HOK HG1.

|  | Replicate 1 (μg) | Replicate 2 (μg) | Replicate 3 (μg) | Average (μg) | Standard Error |
| --- | --- | --- | --- | --- | --- |
| Pertuzumab POK PG1 | 10.6 | 22.575 | 11.193 | 14.78933 | 1.917739 |
| Pertuzumab POK PG1 EAA Supplemented | 21 | 22.213 | 15.95 | 19.721 | 3.896595 |
| Trastuzumab HOK HG1 | 1.0295 | 15.184 | 2.575 | 6.262833 | 1.701694 |
| Trastuzumab HOK HG1 EAA Supplemented | 9.23 | 3.3553 | 5.871 | 6.1521 | 4.48284 |
